# How Many Parasites Species a Frog Might Have? Determinants of Parasite Diversity in South American Anurans

**DOI:** 10.1371/journal.pone.0140577

**Published:** 2015-10-16

**Authors:** Karla Magalhães Campião, Augusto Cesar de Aquino Ribas, Drausio Honorio Morais, Reinaldo José da Silva, Luiz Eduardo Roland Tavares

**Affiliations:** 1 Programa de Pós-Graduação em Ecologia e Conservação, Universidade Federal Paraná, Curitiba, Paraná, Brasil; 2 Faculdade de Computação, Universidade Federal do Mato Grosso do Sul, Campo Grande, Mato Grosso do Sul, Brasil; 3 UNESP—Universidade Estadual Paulista, Campus de Botucatu, Instituto de Biociências, Departamento de Parasitologia, Botucatu, São Paulo, Brazil; 4 Departamento de Biologia, Universidade Federal do Mato Grosso do Sul, Campo Grande, Mato Grosso do Sul, Brasil; Trier University, GERMANY

## Abstract

There is an increasing interest in unveiling the dynamics of parasite infection. Understanding the interaction patterns, and determinants of host-parasite association contributes to filling knowledge gaps in both community and disease ecology. Despite being targeted as a relevant group for conservation efforts, determinants of the association of amphibians and their parasites in broad scales are poorly understood. Here we describe parasite biodiversity in South American amphibians, testing the influence of host body size and geographic range in helminth parasites species richness (PSR). We also test whether parasite diversity is related to hosts’ phylogenetic diversity. Results showed that nematodes are the most common anuran parasites. Host-parasite network has a nested pattern, with specialist helminth taxa generally associated with hosts that harbour the richest parasite faunas. Host size is positively correlated with helminth fauna richness, but we found no support for the association of host geographic range and PSR. These results remained consistent after correcting for uneven study effort and hosts’ phylogenic correlation. However, we found no association between host and parasite diversity, indicating that more diversified anuran clades not necessarily support higher parasite diversity. Overall, considering both the structure and the determinants of PRS in anurans, we conclude that specialist parasites are more likely to be associated with large anurans, which are the ones harbouring higher PSR, and that the lack of association of PSR with hosts’ clade diversification suggests it is strongly influenced by ecological and contemporary constrains.

## Introduction

What determines the number of different species in a given habitat? The search for general laws remains a core issue in community ecology [1]. Parasite ecology is no exception, and parasitologists have dedicated great effort to unveil the laws structuring parasite assemblage [2–5]. By observing how some host species are associated with many parasites while others are not, to assume parasite species richness as a host trait seems a sensible pathway in this pursuit.

One of the main theoretical basis for the study of parasite species richness is the theory of island biogeography. Because parasite communities are formed by colonization and extinction process just like other communities, and because of the insular nature of hosts as habitats, the theory has become popular and influential in parasite community ecology. In this scenario, the rates of parasite colonization and extinction would vary according to features of the hosts [6].

In particular, the body size of the host species is a good potential predictor of parasite species richness (PSR). Large-bodied hosts may provide more space and other resources, and possibly a broader diversity of niches for parasites. Larger hosts live longer, representing less ephemeral habitats than small-bodied species. Thus, larger hosts also have longer exposure to parasites [3]. Similarly, a wider geographical range of the host may result in encounter with and colonization by a greater number of parasite species. Host species ranging over vast areas will overlap with the geographical distribution of several other host species, creating numerous opportunities for host switching [5]. However, the validity of host body size and geographic range as determinants of PSR has been frequently questioned. Unlike islands, hosts can inherit parasites from their ancestors, making it crucial to consider the effect of autocorrelation in comparative analysis across host species [6]. When such corrections are made, the effect of host size and range might lose strength or statistical significance [3, 7].

More recently, with the advance of phylogenetic comparative methods, to consider the evolutionary history of host species as a potential driver of parasite diversity has become increasingly important [8]. Host species vary in their evolutionary time of exposure for acquiring and sharing parasites, and therefore undergo varying co-evolutionary constrains. Furthermore, it has been suggested that not only the richness of parasite species is related to the species richness in host clades, but also that parasites could be potential drivers of host clades diversification through strong selection pressure [9].

A broad view, including ecological and evolutionary mechanisms is needed to understand parasite biodiversity, which can be studied at several scales. As defined by Poulin and Morand [6], “the parasite fauna represent the highest hierarchical level of parasite assemblages; it is composed by all parasite species reported for a given host. The parasite faunas are artificial rather than biological entities, but might be the most relevant scale for macroecological studies”. Here, we investigate the influence of host features relevant to helminth parasite fauna richness in South American amphibians.

Amphibians are very interesting models to study parasite diversity, they comprise a diverse group in terms of taxonomy and life history strategies. Moreover, South America is one of the world′s hotspots of amphibian biodiversity and harbours around 2,599 species [10]. Nonetheless, when we think about quantitative measures or ecological approaches to understand parasite biodiversity, amphibians are the least studied vertebrate group [11, 12]. Here, we use a dataset of published reports of helminth parasites of South American amphibians to: (i) describe parasite biodiversity across hosts linages; (ii) access the nestedness of host-parasite interaction; (iii) test the influence of host body size and geographic range on PSR, correcting the effect of uneven sampling effort and phylogenetic correlation among the hosts; (iv) estimate the amount of sampling effort required to describe amphibian PSR, and how PSR is expected to change with host body size; (v) test if helminth PSR is related to the time of diversification and evolutionary distinctness (contribution of each species to phylogenetic diversity) of South American anurans.

## Materials and Methods

We compiled data on host-parasite interactions from a recent list of helminth parasites of South American amphibians [13]. Two different types of studies constitute this list, the ones focusing on the parasite species (where the known hosts are reported for each parasite), and the ones that focus on particular hosts (all parasites of these hosts are reported). We considered the number of published parasite reports per host our measure of study effort. Only reports that identified host and helminth to species were considered. Because of the shortage in data on other amphibian orders, analyses were carried only with anuran hosts. Data on anurans body size (mean snout vent length) was obtained from papers, field guides and museum assessments. Information on anurans geographic range was compiled from Global Amphibian Assessment database (GAA) [14].

We searched for patterns in species association by evaluating the degree of nestedness in the interaction anurans and their parasites. We adopted the NODF metric [15], and assessed the randomness of matrix nestedness by the analysis of the row–column null model Ce [16]. The calculation of the NODF metric and the simulation of the null model (1000 randomizations) were calculated using the program ANINHADO [16]. All the subsequent analyses were carried out in R 2.14.1 [17]. Host-parasite network is represented with a graph constructed with the package “*igraph*” [18].

We used the Pearson’s correlation test to access associations among the variables in question (study effort, host body size and host geographic rage). To test our main hypothesis, we constructed a non-linear model assuming host size and geographic range as determinants of amphibian PSR. We removed the anuran *Leptodactylus latrans* from analysis because it has been a complex of several cryptic species [10, 19, 20]. It is recognized that the effort dedicated in sampling hosts will determine how well we know parasite diversity. Very frequently, the measure of how intensely hosts have been studied is the best predictor of PSR, making the role of ecological variables, very difficult to detect [6]. Therefore we also considered study effort a determinant of PSR. Nonlinear least squares models relax the requirement of linearity. Then, we first considered an exponential relationship between study effort and PRS, calculated as a Holling type III function [21]. This S-shaped curve is quadratic near the origin, but different from a linear model, it will eventually reach an asymptote. The Holling type III function was calculated as:

$$\begin{array}{c} f\left(x\right)=\frac{ax^{2}}{b^{2}+x^{2}} \end{array}$$ (1)

where *f(x)* is the number of parasites per host, *x* is the number of studies per host, *a* and *b* are the constants. Here, *a* represents the greatest PRS a host can have—the asymptote, and *b* is the number of studies needed to reach it [21]. However, we also expect the PSR to have an exponential relationship with host’s body size and geographic range (as in a Possion regression). Thus, we have:

$$\begin{array}{c} a=e^{c+dy+ez} \end{array}$$ (2)

where *c* is the intercept, *y* is host body size, *z* is host geographic range, and d and e are the respective coefficients. Combining Eqs (1) and (2) we have:

$$\begin{array}{c} f\left(x\right)=\frac{e^{c+dy+ez}x^{2}}{b^{2}+x^{2}} \end{array}$$ (3)

We adjusted this model using the Gauss-Newton algorithm in the “*nls*” function in R.

Another important assumption when making a comparative test is that any values for related species are not truly independent, and treating them as such may lead to pseudoreplication and increased chance of Type I error [2]. Because we consider PSR a host trait, it is necessary to consider that such trait could be inherited from a common ancestor. Therefore, we tested our main hypothesis with an alternative model, a comparative analysis using phylogenetic generalized least squares (PGLS) [22]. The phylogenetic correlation matrix, expects variances and covariances of a continuous trait assuming it evolved under a Brownian model. In the PGLS we first excluded the effect of study effort by calculating a nls with study effort as the predictor variable and PSR as the response variable (Eq 1). We then used the residuals of this nls model as the response variable in the PGLS, and assumed it is determined, additively, by anurans’ body size and geographic range. The correlation among hosts was calculated according to the phylogeny of Amphibia proposed by Pyron and Wiens [23]. We removed all branches of the species that were not in our database of host-parasite interaction. Thus, for the PGLS analysis we only used host-parasite interactions for 118 anuran species, which are included in amphibian’s phylogeny [23]. This analysis were conduced with “*ape*” package [24].

Next, with the same set of data used in the PGLS, we tested the effect of hosts’ evolutionary history in parasite diversity. We first calculated the time of divergence of each species with the function “*sp.ages*” provided by M. Pie (pers. com.). We also calculated the evolutionary distinctness (contribution of each species to phylogenetic diversity) with the function “*ed.calc*” of the “*caper*” package [25] in R. We then tested for the relationship of these two metrics and PSR using the residuals of the nls model as the response variable in a simple linear model.

## Results

### Parasite diversity in anurans

**Fig 1:**
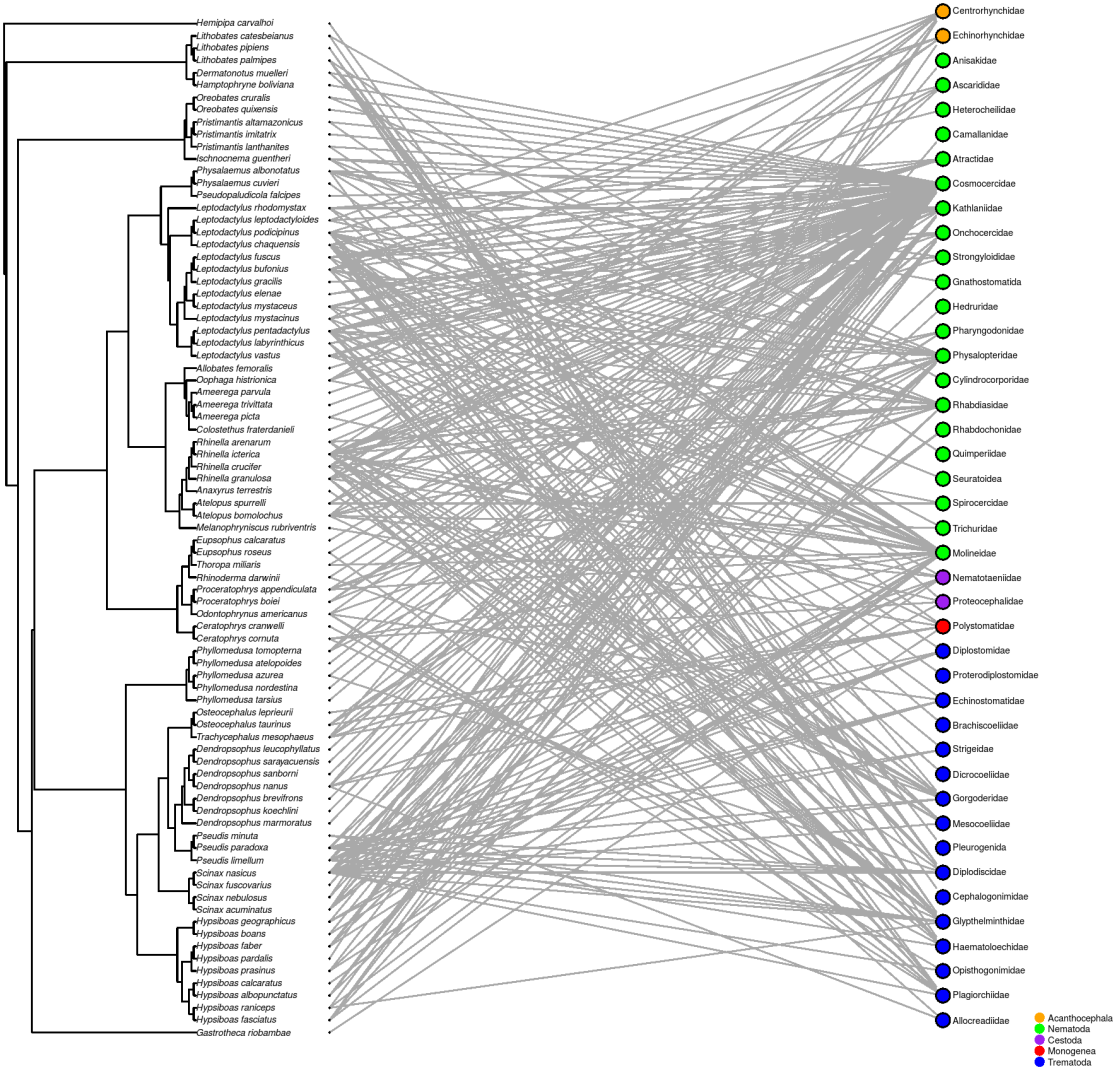
Interacting network of South American anuran species and helminth families. Anuran phylogeny is adapted from [23].

We compiled data of 283 helminth parasites in 180 anuran species, but only 225 helminths and 156 anurans remained after excluding non-specific reports. Nineteen host families are included. Bufonidae, Hylidae and Leptodactylidae are the most representative and account together with almost 60% of the anuran species studied for helminth parasites in South America. Moreover, these are the only host families associated with all helminth major groups (Fig 1).

**Fig 2:**
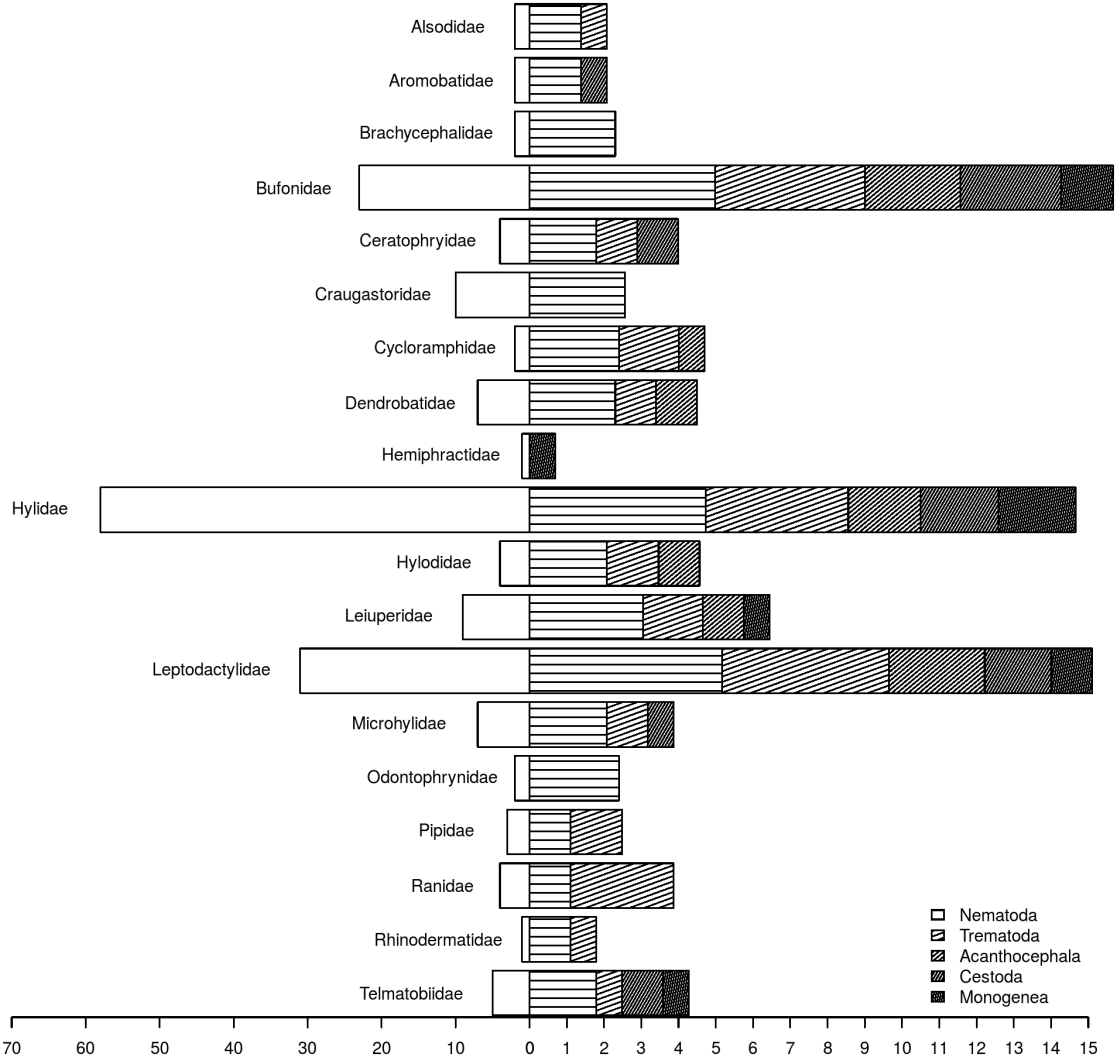
Barplot of helminth parasite species reported to different anuran families. White bars show the number of anuran species surveyed, color bars show the amount (log transformed) of helminth parasites reported for each host family.

We found helminths of the phyla Acanthocephala (two families), Platyhelminthes (two families of Cestoda, one family of Monogenea and of 19 families of Trematoda) and Nematoda (24 families). The most common helminths are nematodes, which occur in practically all host families. Parasites within this group were able to colonize all hosts lineages. Gastrointestinal roundworms of the families Cosmocercidae, Kathlaniidae, Molineidae, Physalopteridae, and lungworms of Rhabdiasidae are the most reported helminths. Trematodes are the second most diverse parasite group and occur in most anuran families, but are more linked to clades of aquatic anurans, such as *Lithobates* and *Pseudis* species (Fig 2). Acanthocephalans, cestodes and monogeneans are less common and more restricted to few anuran species (Fig 2). It is also interesting to note that parasites in groups with fewer species, such as acanthocephalans, cestodes and monogeneans, generally occurred within hosts also parasitized by nematodes and trematodes (Figs 1 and 2). We then tested and found a nested pattern in host-parasite network (*NODF* = 4.46, *P* > 0.01), indicating that specialist parasites are more commonly found in hosts with high PSR.

On average, helminth host range was 3.2 (±4.7, min: 1, max: 34). Out of the 225 helminth species, 113 were restricted to a single host, but the degree of host specificity (host range here) seemed to be not random among helminth taxa. Indeed, all monogeneans are specialists, and 57% of the parasites with a host range of 10 or more are nematodes belonging to the same superfamily (Cosmocercoidea) (Fig 2).

### Determinants of parasite richness

**Fig 3:**
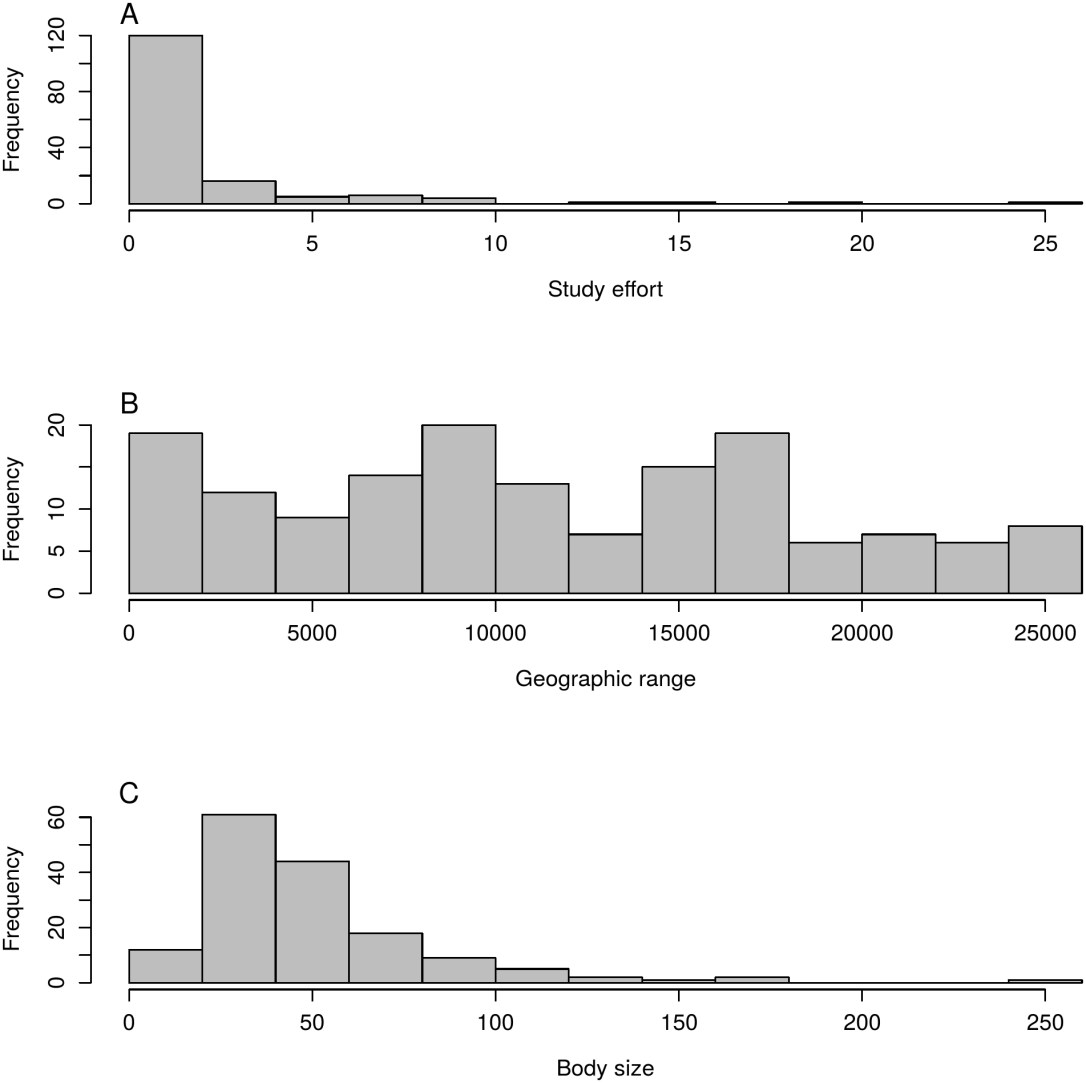
Frequency of the number of studies, body size and geographic range tested as predictors of helminth parasite species richness in South American Anurans.

**Fig 4:**
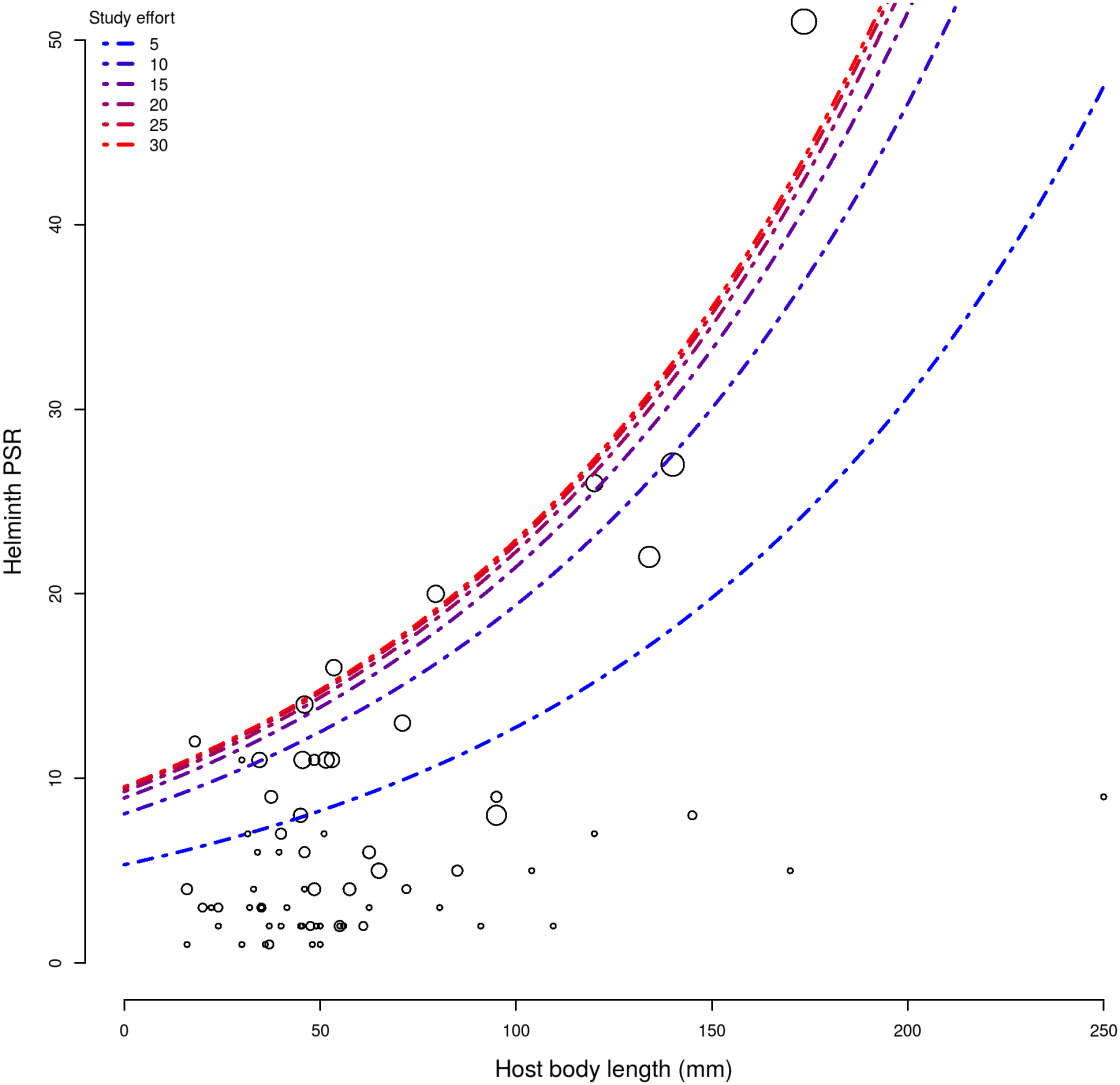
Estimates of helminth parasite species richness (PRS) to South American anurans of different body sizes in response to the number of studies (study effort). Each circle represents an anuran species, the size of each circle shows the real study effort, dashed lines show the estimated PRS in response to different study effort.

The predictor variables we considered as determinants of helminth PSR in anurans—host size, geographic range and study effort are related: large anurans are, in general, both more studied (*r* = 0.44, *p* > 0.01) and widely distributed (*r* = 0.17, *p* = 0.03). Hosts with wider geographic distribution are also more studied (*r* = 0.22, *p* > 0.01) (Fig 3). The nonlinear least square model showed that, as expected, study effort is strongly related to PSR (Table 1). The nls model showed that an average of four studies is needed to reveal 50% of the PSR expected for an anuran host (Table 1). Although, it is important to remember that our dataset, and therefore our predictions, are based in all kinds of studies, including parasite taxonomic reports. Even so, host size is too a good predictor of PSR, and the larger the anuran the richer its parasite fauna is expected to be (Fig 4). Host range, on the other hand, was not significantly related to PSR (Table 1), indicating that larger geographic range of the host does not imply in richer parasite fauna in anurans.

**Table 1:** Results from the nls model for the relationship between study effort, host size and geographic range and parasite species richness in anurans from South America.

Variable	Estimate	Standard Error	t	Pr (> ∣*t*∣)
Intercept	1.1319159	0.8338761	12.809	0.177
Study effort	4.5727182	0.3569919	12.809	< 0.0001
Body size	0.0087551	0.0006565	13.337	< 0.0001
Geographic range	0.1147532	0.0855097	1.342	0.182

Considering the largest geographic range (once it is irrelevant), we can estimate how helminth PSR can increase in response to stronger study efforts (Fig 4). Similarly, we can assume the greatest study effort (26 studies) and estimate mean parasite richness expected for anurans of different body lengths from our dataset (Table 2).

**Table 2:** Estimates of helminth species richness to South American anurans of different body sizes.

Anuran body length (mm)	Expected helminth parasite species richness
30	12.3
50	14.6
70	17.4
90	20.8
110	24.8
130	29.5
150	35.2
170	49.9
190	59.4
210	50.1

The PGLS led us to the same conclusions as the nls. Host size remains significant and host geographic range statistically irrelevant, indicating that the results are consistent after correcting for phylogenetic correlation among hosts (Table 3). Last, we found no association between helminth PSR and host diversification time (*F* = 0.5582, *p* = 0.457) or host evolutionary distinctness (*F* = 0.3896, *p* = 0.5339).

**Table 3:** Results from the phylogenetic generalized least squares (PGLS) for the relationship between study effort, host size, geographic range and parasite species richness in anurans from South America. Model fit parameters are described in the footnote^1^.

Variable	Estimate	Standart Error	t	Pr (> ∣*t*∣)
Intercept	8.9501	11.390	0.7857	0.4339
Body size	0.0543	0.0187	2.8926	0.0047
Geographic range	-0.9743	0.8875	-1.0977	0.2750

^1^AIC = 667.9203, BIC = 678.3007, logLik = -329.9601.

## Discussion

Parasite species account for a great proportion of the planet’s biodiversity, and unveiling this “hidden” diversity is important to a better understand of ecosystem functioning [26]. Among all parasite species, 50.2% are restricted to a single host. Nonetheless, most studies with amphibian helminth assemblages agree about the lack of host specificity often found among these parasites [11, 12, 27, 28]. Data on South American anuran parasites indicate that the low host specificity is quite common, but seems to be restricted to some helminth taxa (Fig 2). Moreover, our description of the diversity of helminth parasites of South American anurans suggests that the distribution of major parasite taxonomic groups among host clades are not random, but largely influenced by host ecology. Further analyses might reveal strong associations between hosts’ life history (i.e. diet and habit) and parasite transmission mode (*i.e* trophic, direct in aquatic habitat, direct in terrestrial habitat).

A good amount of information (23%) on host-parasite interaction was lost after excluding non-specific reports. The lack of taxonomic accuracy is very common when studying invertebrates [29], including the parasitic ones [30]. More specifically, anurans are hosts to a great diversity of larval helminths [13]. This is probably because of the position such vertebrates occupy in ecosystem foodwebs [31, 32]. Because amphibians are prey to several reptile, bird and mammal species, they can act as intermediate or paratenic hosts in the life cycle of several parasite taxa. The precise identification of helminths in such cases is, generally, only possible through molecular biology, which has just recently become used more widely by parasitologists [31, 33]. Therefore, despite the study of parasite diversity having come to a point where there is an amount of data allowing analysis to uncover general patterns, an appealing request for taxonomic studies remains, especially in the tropics [26, 31, 34].

The distribution of specialist parasites amongst hosts was not random, exhibiting a nested pattern. In nested networks, specialist species are more likely to occur in communities with greater species richness [35, 36]. Such structural pattern may decrease competition and increase species coexistence, and contribute to network robustness [37]. The low specificity observed in a representative fraction of the sample may have contributed to the nested pattern of interaction, once low specificity is usually associated with high levels of nestedness and low levels of modularity [38–40].

Examining the determinants of parasite species richness, our results confirmed the strong influence of study effort. The most studied hosts (toads, tree-frogs, and frogs of Bufonidae, Hylidae and Leptodactylidae, respectively) had by far the richest parasite faunas. Some anuran families that seem to have depauperate parasite faunas are, actually, poorly studied. We estimated that an average of four studies is needed to describe 50% of the parasite fauna richness in anurans. Only 22% of South American anurans reached this. However, our dataset includes complete surveys of helminth communities in host populations as well as punctual descriptions and taxonomic reports of helminth species. Therefore, hosts may reach higher PSR with less study effort if they are more targeted of complete parasite surveys. Nonetheless, data on South American anurans (Fig 4) indicate that PSR is still underestimated for most host species.

We found a positive correlation of parasite species richness and host body size for a large dataset of anuran hosts. This result remained consistent after correcting for confounding effects of uneven study effort and hosts phylogenetic correlation. Poulin and Morand [6] and Bush *et al*. [5] state that host body size play a substantial role in the diversification of some parasite fauna, but agreed this importance was far from being universal. Kamiya *et al*. [41] later assume, based on a large interspecific dataset, that the relationship between host body size and PSR is in fact universal across host and parasite taxa, and across levels or scales of study. The underlying mechanism could be that large-bodied hosts may be easier to colonize because of the greater amounts of food they ingest, their large surface area, greater vagility and niche availability [4]. Bush *et al*. [5], Poulin and Morand [6], and Kamiya *et al*. [41] all sum a good amount of evidence of the positive correlation between PSR and body size for a variety of host taxa, but none of them report data on amphibian hosts. Here we add another piece of evidence, for a poorly studied group of hosts, of the role of host size in structuring parasite assemblages.

Different from expected, anurans that are widely distributed geographically do not have, necessarily, richer parasite faunas. Besides promoting geographic overlap with more host species, host range often correlates positively to species abundance and niche breadth [42]. All that could potentially provide more opportunities for colonization of parasites trophically and/or directly transmitted. Indeed, host geographic range is positively related to PSR for fishes, birds and mammals (see the review by Poulin and Morand [6]), and has also been pointed as a universal predictor of PSR [41]. However, we found no effect of host geographic range in determining PSR of South American anurans, whatever the analysis corrected or not for the influence of host’s phylogeny.

Parasite diversity did not correlate to the diversification of hosts’ clades. Nunn *et al*. [9] observed that more rapidly diversifying primate clades had greater parasite diversity, and suggested that parasite diversity may drive hosts’ diversification. Our results do not indicate that long evolutionary time of exposure imply in greater parasite diversity, and also that helminth parasites might not exert strong selective pressure to drive divergence among anuran populations. Furthermore, there are other biogeographical and contemporary constrains that are influential to parasite diversity. For instance, host species occurring close to the equatorial region might have greater parasite diversity [8]. Additionally, overall free-living diversity, which includes greater availability of potential intermediate host species, has been proven drivers of parasite diversity [43–45].

Overall, we found that nematodes are the most common anuran parasites, and specialist helminth taxa are generally associated with larger hosts that harbour the richest parasite faunas. Anurans body size determines PSR, the larger the anuran the richer the parasite fauna. Considering both the structure and the determinants of PRS in anurans, specialist parasites are more likely to be associated with large hosts.

## Supporting Information

S1 Dataset Body size, geographic range, number of studies and helminth parasites reported for South American anurans. (XLSX)
